# Keeping busy learners informed: email is most useful for medical residents!

**Published:** 2019-07-24

**Authors:** Anna Karwowska, Sandy Tse

**Affiliations:** 1 Division of Emergency Medicine, Department of Pediatrics, University of Ottawa, Ontario, Canada

## Abstract

**Background:**

Educators need to ensure trainees have access to the rotation orientation information they need in the most effective way possible. We wanted to find the best method to distribute this information.

**Methods:**

We provided post-graduate medical trainees rotating through the Pediatric Emergency Medicine program at the Children’s Hospital of Eastern Ontario in Ottawa, Canada, the regular rotation information package three ways: email, online, and paper. We surveyed them to find out which method(s) they used and which they found most useful.

**Results:**

All trainees were able to access the electronic orientation package via email and most found this method useful. Most also found the paper package distributed at the orientation helpful. Few accessed the online wiki site.

**Conclusion:**

Using email is efficient and effective and can save both the time and cost of producing paper packages. The wiki site was not used frequently in our cohort, but may be worth future exploration.

## Introduction

Medical training programs must provide residents with appropriate information about rotation-specific objectives, schedules and other relevant rotation requirements.^1^ Usually, this has been accomplished via a paper package at a first-day rotation orientation session. A previous study showed trainees used technology to access medical information.^2^ Mobile technologies can enhance learning activities for all levels of trainees.^3^ Therefore educators should seek alternative options to ensure trainees have access to the information they need in the easiest and most effective way possible. We did not find a study that evaluated how post-graduate medical trainees prefer to access orientation information.

Paper has been the most common tool used to distribute orientation information, however it is unclear if trainees read the hard copies when they tend to carry most of their information around in their tablets and cellphones. Emailing information packages is an obvious option as it is popular, quick and cost effective. However, “email overload” is a reality and important emails with essential information may not be opened or fully perused by the intended recipient.^4^ For both the sender and recipient, this can affect rotation experience and satisfaction. Wiki websites facilitate the sharing of information online, and allow users access to a central repository regardless of geographical location.^5^ Some considered them a good platform for on-demand information access.^6^ However, this technology requires online access, and the user needs to navigate through the site to find the required information. This multi-step process may deter trainees from accessing this resource.

We studied the distribution of rotation information to training residents in the Children’s Hospital of Eastern Ontario (CHEO) Pediatric Emergency Medicine rotation in Ottawa, Canada. This is both a descriptive inventory of medical trainee opinion of utility of email, paper and wiki methods of information distribution, as well as a quality assurance of satisfaction of access to rotation information packages.

## Methods

The study was approved by the CHEO Research Ethics Board. Completion of the survey implied consent to participate in the study.

In our prospective survey of medical trainees rotating through the CHEO Pediatric Emergency training program, we provided the information package three ways:

1. An email was sent to each resident, approximately one week prior to the start of rotation, inviting them to the orientation session and providing attachments with orientation information.
2. A wiki website was created to act as a repository for the same information package. The wiki also contained an electronic presentation that visually oriented learners to the department and some of its processes. The link was sent with the email.
3. A paper package containing the same information package as in the email attachments and on the wiki site was provided at the mandatory orientation session.

All 71 trainees attending the mandatory orientation session at the start of their first CHEO Pediatric Emergency rotation were approached to complete the survey. We surveyed eligible residents over five 4- week-block periods.

The survey had 12 questions regarding demographic information (year of training, age range), overall satisfaction with how respondents received their orientation information and which method they used to access the information. Responses were anonymous.

We collected and collated the surveys then entered the data into *Excel* and calculated the descriptive statistics (frequencies and percentages).

## Results

The survey response rate was 73.2% (52/71) of eligible residents. Most respondents (33) were 26-30 years of age; six each were in the 20-25, 31-35 and 35- 40 ranges, and one was over 40. Thirty-six were in post-graduate Year 1 of training and eight each in their second and third years. One hundred per cent of respondents (52) had computers at home, 59.6% (31) had tablets and 94.2% (49) had smartphones. Although 52 residents responded, not all answered every question.

The majority (48 of 51 respondents) read some or all of the orientation information on the email and three read a little of it. Twenty-four of 49 respondents read some, all or a little of the information on the wiki site; only 10 of those viewed the orientation video presentation.

Both the email and paper packages methods of distribution were found somewhat to very useful by 46 of 50 respondents. Of 29 respondents who accessed the wiki, 23 found that method to be somewhat to very useful (See Table 1).

**Table 1:** Rating of usefulness of methods providing orientation information

	Paper orientation n=50 (%)	Email orientation n=46 (%)	Wiki site orientation n=29 (%)
**Not useful**	4 (8)	0	6 (20.7)
**Somewhat useful**	18 (36)	10 (20)	3 (10.3)
**Useful**	17 (34)	25 (50)	17 (58.6)
**Very useful**	11 (22)	11 (22)	3 (10.3)

## Discussion

All residents had computer access at home and most were also able to access electronic information via smart phone, suggesting that there are no accessibility barriers to distribution of information electronically. Most residents reported reviewing orientation information when it was sent to them via email prior to the start of the rotation. This would suggest that less time could be spent in reviewing this information in the face-to-face orientation session on day 1 of the rotation.

Despite having seen it via email, most also found the paper package provided on orientation day useful. Perhaps it provides a physical reminder of the email package, or perhaps because this hard copy was available for the first day orientation, the residents used this document when information was reviewed on site. It is unknown at this time if the trainees would miss having the paper package if it was not provided, but program administrators could save time and resources if trainees only needed a digital copy. In fact, trainees could review the information digitally prior to or on day 1, print copies for themselves if needed and also easily share with colleagues if someone did not have their copy available.

The information provided on the wiki site was less likely to be used by respondents. The factors that may have affected this response may include the following: 1) duplication of the material already visible on email attachments, 2) extra steps to access an online platform, 3) lack of time to explore the wiki site, or 4) data costs to use the online platform. We also observed that few trainees reported viewing the video orientation presentation available online. There may be time constraints for trainees while working on another rotation to do this, or perhaps they considered this another duplication of the upcoming face-to-face orientation. Interestingly, despite the fact that there was no additional information specific to the wiki site that was not provided elsewhere, 49.5% of respondents did access the online information and there may be utility to house a reservoir of rotation specific requirements there in the future. There is potential on this platform to include larger files, video instruction and other resources for rotation specific needs.

While we did ask which of the three methods of information distribution the residents found useful, we did not ask which one they preferred or why. These are important questions for future research.

### Conclusion

There are no accessibility issues for electronically distributed information, and trainees are comfortable with available technology. Email can be used to provide needed information, saving both the time and cost of producing paper packages. The efficient use of wiki sites is still developing. Future studies could look at how effective it would be if, instead of attachments, an information email simply provided a link to a wiki site.
